# Convalescent Homes Association

**Published:** 1907-03-02

**Authors:** W. S. Chursk, M. O. FitzGerald, S. H. Habershon

**Affiliations:** Chairman; Hon. Sec.; Hon. Sec.


### Editor's Letter-Box.

[Our Correspondents are reminded that prolixity is a great bar tc publication, and that brevity of style and conciseness of statement greatly facilitate early insertion.]

### CONVALESCENT HOMES ASSOCIATION.

*To the Editor of* The Hospital.

Sir,—About two years ago a public appeal was made representing the great need of further accommodation for convalescents recovering from surgical operations and from special medical ailments. The Convalescent Homes Association discouraged any outlay on bricks and mortar, and made efforts to induce certain convalescent homes to adapt themselves to the admitted needs of hospital, which have been abundantly successful, and have resulted in the opening of some forty-one new surgical beds at existing homes.

These beds can only be obtained at a cost of from 10s. to 7s. 6d. per week for adults and of 6s. per week for children.

Information regarding available beds and all other convalescent matters can be supplied at the new Inquiry Bureau opened by the Convalescent Homes Association at 32 Sackville Street, Piccadilly, W. A small fee is charged. It is hoped that the public will avail themselves of this new and useful means of gaining information as to the provision made for convalescents who are not usually taken in by convalescent homes.

It seems probable that this new provision of surgical beds is not fully known, and the special attention of sisters of wards is directed to the novel effort of the Convalescent Homes Association; for, in spite of the urgent cry for such beds, now that they have been provided they are not filled. We have little doubt that the need exists, but there is no question that the demand is not so acute as has been represented in some quarters.

One fact has been elicited by the work of the Convalescent Homes Association—namely, that there is a pressing want of beds for children suffering from chronic suppuration and surgical wounds—cases which block the wards of our hospitals and which are at present very inadequately provided for.

The Convalescent Homes Association has recently made an attempt to obtain from the Parks Committee of the London County Council one or more of their mansions in public parks for this purpose, but without success. The appeal of the Lord Mayor does not quite touch the case of chronic surgical ailments in children. It is rather made for an equally needy class—those children who are crippled and deformed as the results of past disease. Admirable as the effort is, and thoroughly as it deserves success, it does not touch the class of children who are suffering from chronic diseases, convalescent, though not yet healed. The Convalescent Homes Association have made, and are still making, an attempt to relieve these. We are, sir,

Your faithfully,

W. S. Churck (Chairman).

M. O. FitzGerald

S. H. Habershon } (Hon. Secs.).

